# Chemotherapy-induced hyaluronan production: a novel chemoresistance mechanism in ovarian cancer

**DOI:** 10.1186/1471-2407-13-476

**Published:** 2013-10-14

**Authors:** Carmela Ricciardelli, Miranda P Ween, Noor A Lokman, Izza A Tan, Carmen E Pyragius, Martin K Oehler

**Affiliations:** 1 Discipline of Obstetrics and Gynaecology, School of Paediatrics and Reproductive Health, Research Centre for Reproductive Health, Robinson Institute, University of Adelaide, Adelaide 5005, South Australia, Australia; 2 Research Centre for Infectious Diseases, School of Molecular Biosciences, University of Adelaide, Adelaide 5005, South Australia, Australia; 3 Department of Gynaecological Oncology, Royal Adelaide Hospital, Adelaide 5005, South Australia, Australia

**Keywords:** Extracellular matrix, Hyaluronan, Chemotherapy, ABC transporters, Ovarian cancer

## Abstract

**Background:**

Hyaluronan (HA) an important component of the extracellular matrix, has been linked to tumor progression and drug resistance in several malignancies. However, limited data is available for ovarian cancer. This study investigated the role of hyaluronan (HA) and a potential link between the HA-CD44 pathway and membrane ATP binding cassette (ABC) transporter proteins in ovarian cancer chemoresistance.

**Methods:**

We investigated the ability of HA to block the cytotoxic effects of the chemotherapy drug carboplatin, and to regulate the expression of ABC transporters in ovarian cancer cells. We also examined HA serum levels in ovarian cancer patients prior to and following chemotherapy and assessed its prognostic relevance.

**Results:**

HA increased the survival of carboplatin treated ovarian cancer cells expressing the HA receptor, CD44 (OVCAR-5 and OV-90). Carboplatin significantly increased expression of *HAS2*, *HAS3* and *ABCC2* and HA secretion in ovarian cancer cell conditioned media. Serum HA levels were significantly increased in patients following platinum based chemotherapy and at both 1st and 2nd recurrence when compared with HA levels prior to treatment. High serum HA levels (>50 μg/ml) prior to chemotherapy treatment were associated with significantly reduced progression-free (P = 0.014) and overall survival (P = 0.036). HA production in ovarian cancer cells was increased in cancer tissues collected following chemotherapy treatment and at recurrence. Furthermore HA treatment significantly increased the expression of ABC drug transporters (*ABCB3, ABCC1, ABCC2,* and *ABCC3*), but only in ovarian cancer cells expressing CD44. The effects of HA and carboplatin on ABC transporter expression in ovarian cancer cells could be abrogated by HA oligomer treatment. Importantly, HA oligomers increased the sensitivity of chemoresistant SKOV3 cells to carboplatin.

**Conclusions:**

Our findings indicate that carboplatin chemotherapy induces HA production which can contribute to chemoresistance by regulating ABC transporter expression. The HA-CD44 signaling pathway is therefore a promising target in platinum resistant ovarian cancer.

## Background

Ovarian cancer is the most lethal gynecological cancer and ranks as the fourth most common cause of cancer-related death in women in the Western world [1]. The standard treatment for advanced ovarian cancer is debulking surgery, followed by platinum-based chemotherapy. This standard treatment results in a complete response rate of 40-60%, however, more than 90% of these patients relapse after 2 years [2]. Recurrent ovarian cancer in most cases becomes incurable due to the development of chemoresistance.

Chemoresistance is multifactorial in nature involving tumor-related and drug-related factors, as well as interactions with the tumor microenvironment itself. A well established cause involves increased expression of members of the membrane efflux ATP binding cassette (ABC) transporter family, which decrease the intracellular accumulation and retention of chemotherapy drugs [3]. ABC transporters are a family of membrane proteins that transport a wide range of substrates, including metabolic products, nutrients, lipids, and drugs, across extracellular and intracellular membranes, and have been shown to play an important role in many human diseases [4,5]. Phylogenetic analysis places the 48 known human ABC transporters into 7 distinct subfamilies (ABCA-ABCG) [4]. The first ABC transporter identified was MDR1 (ABCB1), also known as p-glycoprotein [6]. ABCB1 plays a critical role in drug fluxes and chemoresistance in many malignancies, including ovarian cancer [7-10]. Other ABC transporters, including ABCB3 (TAP2), ABCC1 (MRP1), ABCC2 (MRP2, cMOAT), and ABCC3 (MRP3), have been shown to be involved in ovarian cancer chemoresistance [11-15].

A component of the tumor microenvironment linked to chemoresistance is the extracellular matrix (ECM) molecule, hyaluronan (HA) [16]. HA is a large polysaccharide that is assembled into pericellular and ECM in many tissues [17]. HA has a role in various cell functions such as adhesion, motility, and differentiation. It has also been implicated to be a key player in cancer metastasis [17,18]. Many human tumors, including ovarian cancer, are surrounded by a connective tissue matrix enriched with HA [18-20]. Increased HA has been shown to be an independent predictor of ovarian cancer survival [19]. HA levels significantly correlate with the degree of invasiveness and metastatic potential in malignant ovarian tumors [19,21], and it promotes the attachment of cancer cells to peritoneal cells via interactions with its major surface receptor, CD44 [22-25].

HA has been shown to reduce the ability of chemotherapy drugs to cause cancer cell death in a variety of malignancies [26-29]. Furthermore, several studies have demonstrated that HA interactions with CD44 can increase resistance to numerous chemotherapy drugs and increase the expression of the ABC drug transporters [28,30-34]. The co-localization of CD44 with ABCB1 and ABCC2 in ovarian cancer tissues suggests a functional link between CD44 expression and chemoresistance [35]. However, knowledge regarding the importance of HA-CD44 interactions in mediating chemoresistance and regulating ABC transporter expression in ovarian cancer is limited. In this study we therefore assessed the ability of HA to block the cytotoxic effects of the chemotherapy drug carboplatin (CBP) on ovarian cancer cells and investigated a potential link between the HA-CD44 pathway and ABC transporter expression. To relate findings with the clinical situation, we measured HA serum levels in ovarian cancer patients at diagnosis, during chemotherapy and at recurrence and determined the relationship with patient outcome.

## Methods

### Cell lines

The human ovarian cancer cell lines OVCAR-3, SKOV-3, and OV-90 were obtained from American Type Culture Collection (ATCC, Manassas, VA, USA). OVCAR-5 cells were obtained from Dr. Thomas Hamilton (Fox Chase Cancer Center, Philadelphia, PA) [36]. All ovarian cancer cell lines were maintained in RPMI 1640 medium supplemented with 4 mM L-glutamine and antibiotics (100 U penicillin G, 100 μg/ml streptomycin sulfate and 0.25 μg/ml amphotericin B, Sigma Aldrich, St Louis, MO, USA) at 37°C in an environment of 5% CO_2_ as described previously [37].

### Cell survival assays

Cells were plated at 5,000 cells per well, grown for 24 hr, and then treated with CBP (0–300 μM, Mayne Pharma, Victoria, Australia) for 72 hr in normal growth media. Cell survival was calculated by MTT assay, as per the manufacturer’s instructions (Sigma Aldrich). The inhibitory concentration (LD_50_) of CBP was calculated from three independent experiments performed in triplicate using exponential regression curve fitting. To assess the effect of HA on cell survival, cells were plated at 5,000 cells per well, grown for 24 hr, and then treated with IC_50_ dose of CBP and HA (0–100 μg/ml, H1504, Sigma Aldrich) ± CD44 neutralizing antibody (Clone A020, 20 μg/ml Calbiochem, NSW, Australia), control IgG (20 μg/ml, BD Biosciences, North Ryde, NSW, Australia) or HA oligomers (HYA-OLIGO 6–10, 10–250 μg/ml, North Star Bioproducts, Associates of Cape Cod Inc, East Falmouth, MA, USA or 10-250 μg/ml HYA-OLIGO 8, Hyalose LLC, Oklahoma City, OK, USA) for 72 hr before cell survival assessment by MTT assay. Chemosensitivity of SKOV-3 cells to CBP in presence of HA oligomers (250 μg/ml) was determined using the MTT assay as described above. Exponential curve fitting was used to the calculate equation of the line and the CBP LD_50_ in the presence of CBP alone and combined with HA oligomers.

### Quantitative real-time PCR

Cells were plated at 5,000 cells per well for 24 hr, and then treated with control medium, CBP LD_50_ dose or HA (5 μg/ml) for 72 hr. Cells were also treated CBP or HA in the presence or absence of HA oligomers (10-250 μg/ml HYA-OLIGO 6-8, Hyalose LLC). Total RNA was isolated from ovarian cancer cell lines and reverse transcribed using the TaqMan® Gene expression Cells-to-CT™ kit (Applied Biosystems, Mulgrave, Victoria, Australia), as per the manufacturer’s instructions. Briefly, lysis solution with DNAse was added to each well and incubated for 5 min at room temperature. Stop solution was then added to each well and mixed. The lysate (10 μl) was added to a 40 μl reverse transcription master mix and reverse transcribed for 1 hr. Resultant cDNA was stored as 50 μl aliquots at −20°C for qRT-PCR analysis.

qRT-PCR reactions were performed on triplicate samples using TaqMan® primer sets for genes of interest, as detailed in Additional file 1: Table S1 using the 7900HT Fast Real-Time PCR System (Applied Biosystems, Mulgrave, Victoria, Australia). Briefly, PCR reactions were made up to 10 μl and contained TaqMan® Gene Expression Master Mix (2×), primers for the gene of interest, nuclease free water, and the sample cDNA. PCR cycling conditions were as follows: 50°C for 2 min, 95°C for 10 min (with 40 cycles following of 95°C for 15 sec), and 60°C for 1 min. CT values were normalised to the house keeping gene β-actin and calibrated to no treatment control using the 2^-∆∆CT^ method.

### HA ELISA assay

An HA ELISA kit (DY3614, R&D Systems, Minneapolis, MN) was used to determine the concentration of HA in serum samples or conditioned media (CM), as per manufacturer’s instructions. Serum was assessed from ovarian cancer patients at diagnosis (n = 101), after chemotherapy (n = 52), at 1^st^ recurrence (n = 17) and 2^nd^ recurrence (n = 5). Matched serum samples at diagnosis and following at least two cycles of chemotherapy were available from 32 patients. Serum samples were also obtained from patients with benign ovarian tumors (n = 22) and healthy controls (n = 27). All samples were collected with approval from the Royal Adelaide Hospital Human Ethics Committee and informed written consent was obtained from all participants involved in this study. CM was collected from cells (OVCAR-5, OVCAR-3, OV-90, and SKOV-3) plated at 5,000 cells per well and grown for 24 hr before 72 hr treatment with their IC_50_ concentration of CBP or control media. The detection limit of the assay was 100 pg/ml, and the coefficient of variation between assays was 9.21%.

### HA staining in ovarian cancer tissues

Formalin fixed tissue was obtained from ovarian cancer patients at surgery (n = 10), postchemotherapy (n = 9) and at recurrence (n = 4). Tissues samples were collected with approval from the Royal Adelaide Hospital Human Ethics Committee. Informed written consent was obtained from all participants involved in this study. Tissue sections (5 μm) were mounted onto positively charged slides (SuperFrost VR Plus, Menzel-Glaser, Braunschweig, Germany) and heated at 60°C for 1.5 hr. HA in ovarian cancer sections was detected using HABP (2 μg/ml, Seikagaku Corp, Japan) [38]. The intensity of HA in the epithelial and stromal compartments was assessed using a manual scoring method: strong (3+), moderate (2+), weak (1+), or negative (0). A score of 0 or 1+ was defined as low HA staining and a score or 2+ or 3+ was defined as high HA staining. The % of HA positive cancer cells were independently assessed in the three tissue groups.

### HA staining and ABCC2 immunocytochemistry

OVCAR-5 cells (2×10^4^ cells/well) were plated in 8 well tissue culture chamber slides (Nunclon™ Lab-Tek II Chamber slide, RS Glass Slide, Naperville, IL) in 500 μl 10% FBS RPMI for 24 hr and treated for 72 hr with IC_50_ concentration of CBP or control media. OVCAR-5 cells were fixed with cold 100% methanol (5 min) and cold 100% acetone (3 min), washed with PBS and blocked with 5% donkey serum and incubated overnight with biotinylated HABP (2 μg/ml, Seikagaku Corp, Japan) and/or mouse ABCC2 monoclonal antibody (1/25, Clone M2I-4, Abcam, Cambridge, United Kingdom). HA and ABCC2 were visualized with Cy3-conjugated streptavidin (1/100, 1 hr at RT, catalogue no: 016-160-084, Jackson Immunoresearch Laboratories, West Grove, PA, USA) or FITC-conjugated AffiniPure donkey anti-mouse (1/100, 1 hr at RT, catalogue no: 715-096-151, Jackson Immunoresearch Laboratories), respectively. Nuclei were stained with 4′,6′-diamidino-2-phenylindole dihydrochloride solution (DAPI, Molecular Probes, Eugene, OR, USA) and slides were mounted with fluorescent mounting medium (Dako Labs, Glostrup, Denmark). Cells were viewed with an epifluorescence microscope (BX50, Olympus Australia) and imaged using a 20× objective and a Spot RT digital camera (Diagnostic Instruments, Sterling Heights, MI). Negative controls included only Cy3-conjugated streptavidin and FITC-conjugated AffiniPure donkey anti-mouse and also *Streptomyces* hyaluronidase (30 min RT, 10 U/ml, Sigma-Aldrich) treatment for the HA staining.

### Statistical analyses

All analyses were performed using the PASW 17.0 Statistical software (SPSS Inc., Chicago, IL). The Mann–Whitney U, Kruskal-Wallis, Wilcoxon signed rank or Chi Square tests were used to determine statistical significance between patient groups. For cell line studies, the Student’s t-test and one way ANOVA test with the Tukey or Dunnett C post-hoc tests were used to determine statistical significance between control and treatment groups. Clinical follow-up data for disease progression and survival was available for 77 and 83 ovarian cancer patients respectively. All patients received either CBP alone or a combination of CBP and paclitaxel. Forty five percent (35/77) of the patient’s progressed and 27.7% (23/83) died from ovarian cancer at the time of census (1^st^ December 2012). Kaplan-Meier and Cox regression analyses with progression or death due to ovarian cancer was used as the endpoint to determine whether HA levels prior to chemotherapy treatment were related to progression-free survival (PFS) or overall survival (OS). Statistical significance was accepted at *P* < 0.05.

## Results

### HA treatment increases ovarian cancer cell resistance to carboplatin

**Figure 1:**
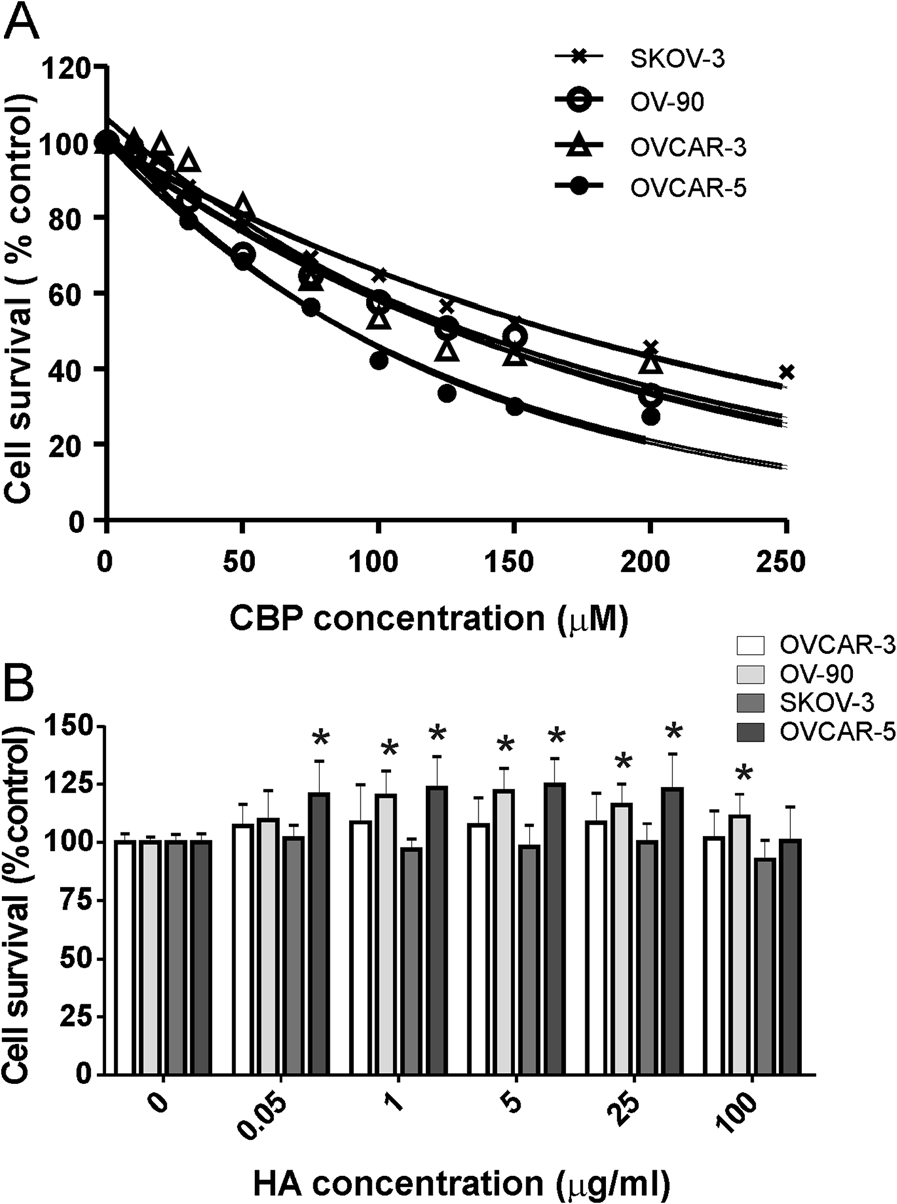
**HA increases ovarian cancer cell resistance to carboplatin. A**. CBP dose response growth curves for ovarian cancer cell lines. LD_50_ for SKOV3, OV-90, OVCAR-3 and OVCAR-5 was calculated using exponential curve fitting. **B**. Cell survival of ovarian cancer cells treated with CBP (LD_50_ dose) ± HA (0–100 μg/ml) after 72 hr. HA treatment increased the survival of OVCAR-5 and OV-90 cells, but not of SKOV-3 and OVCAR-3 cells following treatment with CBP (LD_50_ dose). Data are expressed as percentage of no CBP control, mean ± SEM from 3–5 independent experiments performed in triplicate. *, significantly different from control (*P* < 0.05, independent t test).

**Figure 2:**
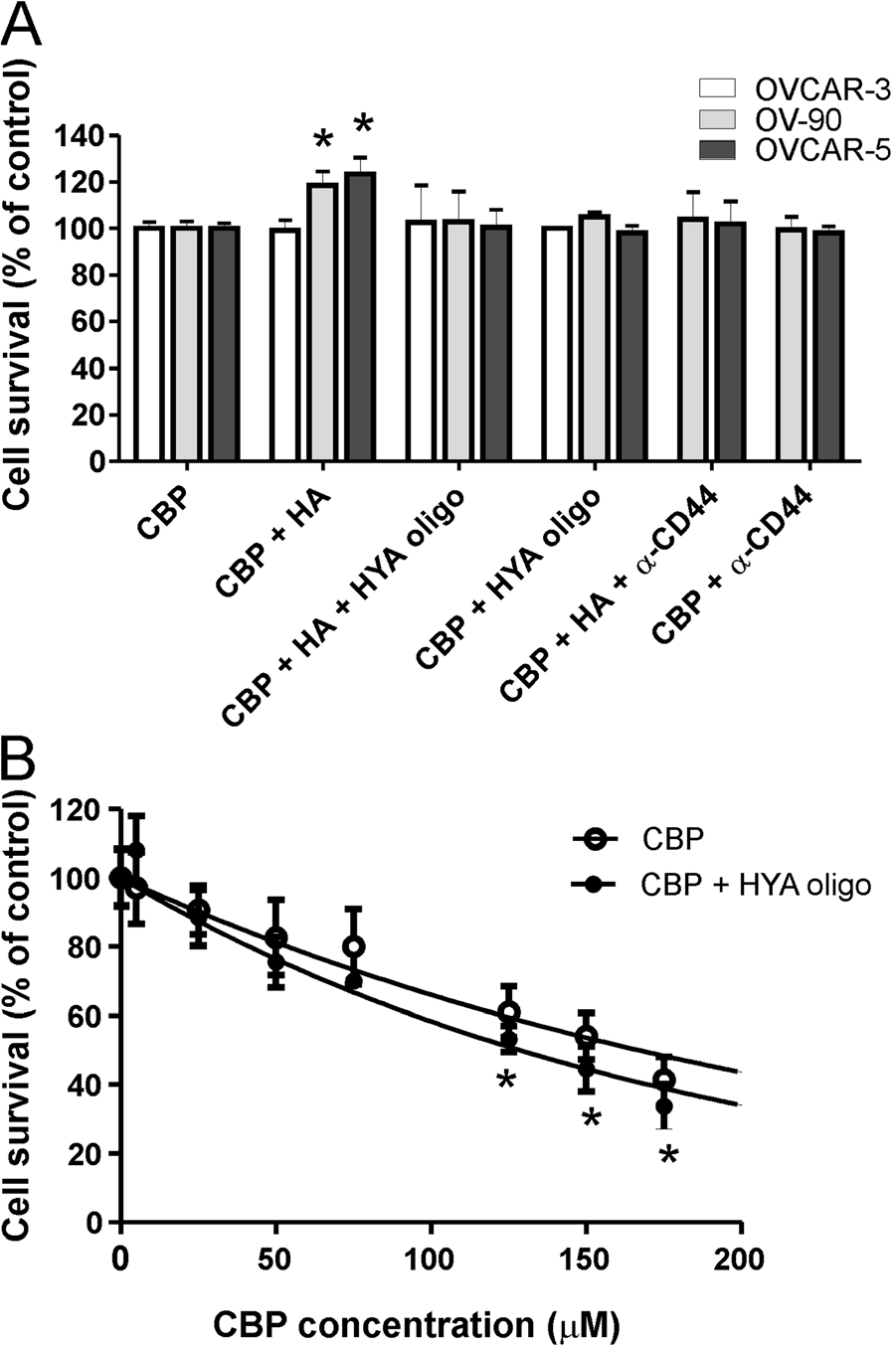
**HA-CD44 interactions mediate chemoresistance in ovarian cancer cells. A**. HA oligomers (HYA oligo, 250 μg/ml) and anti-rat neutralizing CD44 monoclonal antibody (20 μg/ml) blocked increased survival of OVCAR-5 and OV-90 cells treated with HA (5 μg/ml) and carboplatin (CBP, LD_50_ dose), but had no effect on OVCAR-3 cells. **B**. HYA oligo increased the sensitivity of SKOV-3 cells to CBP. SKOV-3 cells were treated with CBP (0–200 μM) ± HYA oligo (250 μg/ml) for 72 hr. The CBP LD_50_ calculated using exponential curve fitting was 173 μM in the presence of CBP alone and reduced to 123 μM when CBP was combined with HYA oligo. Data are expressed as percentage of control, mean ± SD from 2 independent experiments performed in triplicate. *, significantly different from control (*P* < 0.05, independent t test).

To determine whether HA contributes to chemoresistance, we assessed the ability of HA to block the cytotoxic effect of CBP. We determined the CBP LD_50_ dose for a range of ovarian cancer cell lines with increasing invasive capacity. The SKOV-3 cells were the most resistant to CBP (LD_50_ = 184 μM), followed by OV-90 (LD_50_ = 145 μM), OVCAR-3 (LD_50_ = 113 μM) and OVCAR-5 (LD_50_ = 81 μM, Figure 1A). We assessed cell survival following treatment with both CBP + HA or CBP alone using the LD_50_ CBP dose determined for each cell line. HA treatment increased OVCAR-5 and OV-90 cell survival, but did not affect the survival of SKOV-3 or OVCAR-3 cells (Figure 1B). As little as 0.01 μg/ml HA and 0.05 μg/ml HA were able to significantly increase survival of OVCAR-5 and OV-90 cells, respectively. HA treatment alone (0.05-100 μg/ml) did not affect cell proliferation of any of the ovarian cancer cell lines however higher concentration of HA (500 μg/ml) significantly inhibited growth of OVCAR-5 and SKOV3 cells (Additional file 2: Figure S1). The increased cell survival (OVCAR-5 and OV-90) observed with CBP and HA treatment was significantly blocked by either the addition of HA oligomers or a neutralising CD44 antibody (Figure 2A). HA oligomers had no effect on the survival of OVCAR-3 (Figure 2A). Importantly, HA oligomers significantly increased the sensitivity of chemoresistant SKOV3 ovarian cancer cells to CBP. Combined treatment with CBP and HA oligomers, significantly reduced SKOV3 survival and reduced the CBP LD_50_ from 173 μM to 123 μM (Figure 2B).

### Chemotherapy treatment increases HA production and ABCC2 expression by ovarian cancer cells

**Figure 3:**
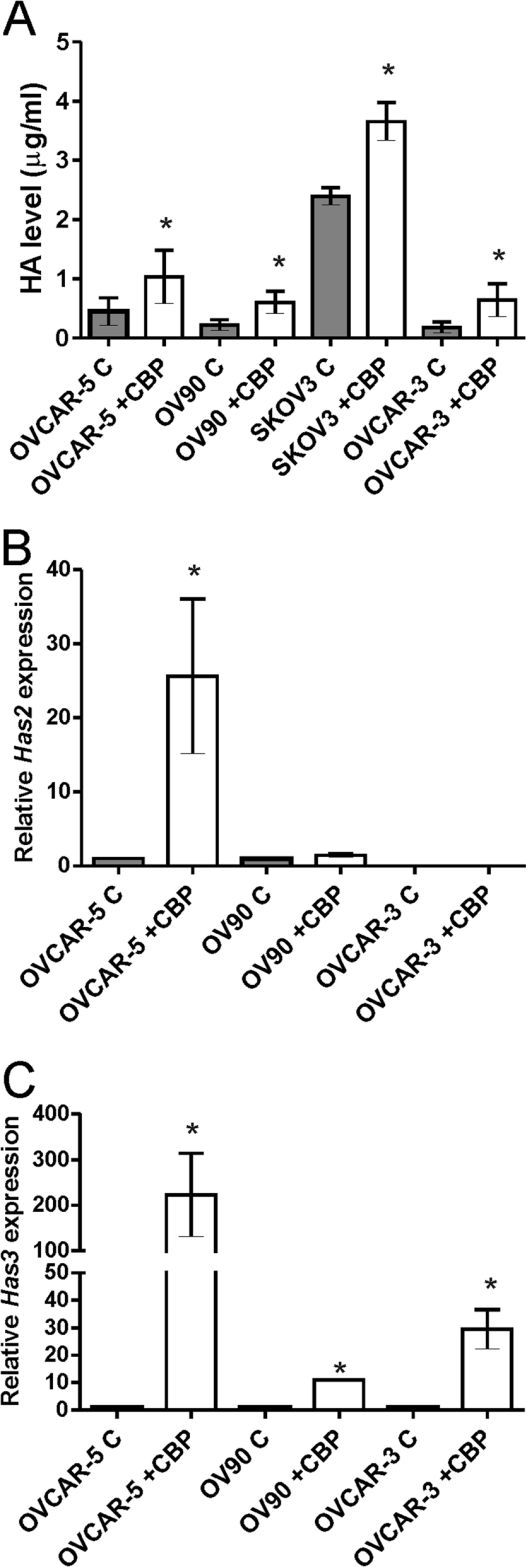
**Carboplatin treatment increases HA production by ovarian cancer cells. A**. HA levels in the CM of ovarian cancer cell lines, determined by ELISA assay. Data represent mean ± SEM from 3 independent experiments performed in duplicate. *, significantly different from no CBP control (*P* < 0.05, independent t test). **B**. qRT-PCR for *HAS2* expression. **C**. qRT-PCR for *HAS3* expression. Relative gene expression in **B** and **C** was determined by calibration against no CBP control and normalized to the house keeping gene β-actin using the 2^-∆∆CT^ method. Data represents mean ± SEM from 2 independent experiments performed in triplicate. *, significantly different from no CBP control (*P* < 0.05, independent t test). C = no CBP, CBP = carboplatin (LD_50_ dose), CM = conditioned media.

We investigated whether CBP increases HA production and the expression of ABCC2, an ABC transporter shown to confer ovarian cancer cell resistance to platinum based chemotherapy agents including CBP [13,39]. Treatment with the LD_50_ CBP dose significantly increased HA secretion in the CM of OVCAR-5 (3.7 fold), OVCAR-3 (4.1 fold), SKOV-3 cells (1.6 fold), and OV-90 cells (2.6 fold) (*P* < 0.05) (Figure 3A). SKOV-3 cells, the most CBP-resistant cell line, produced the highest levels of HA. Increased *Has2* and *Has3* expression was observed in OVCAR-5 cells treated with CBP (6 and 44 fold, respectively, Figure 3B and 3C). *Has3* (Figure 3C), but not *Has2* (Figure 3B), expression was increased in OV-90 and OVCAR-3 cells following treatment with CBP. HA levels in CM correlated with *Has2* and *Has3* gene expression (data not shown).

**Figure 4:**
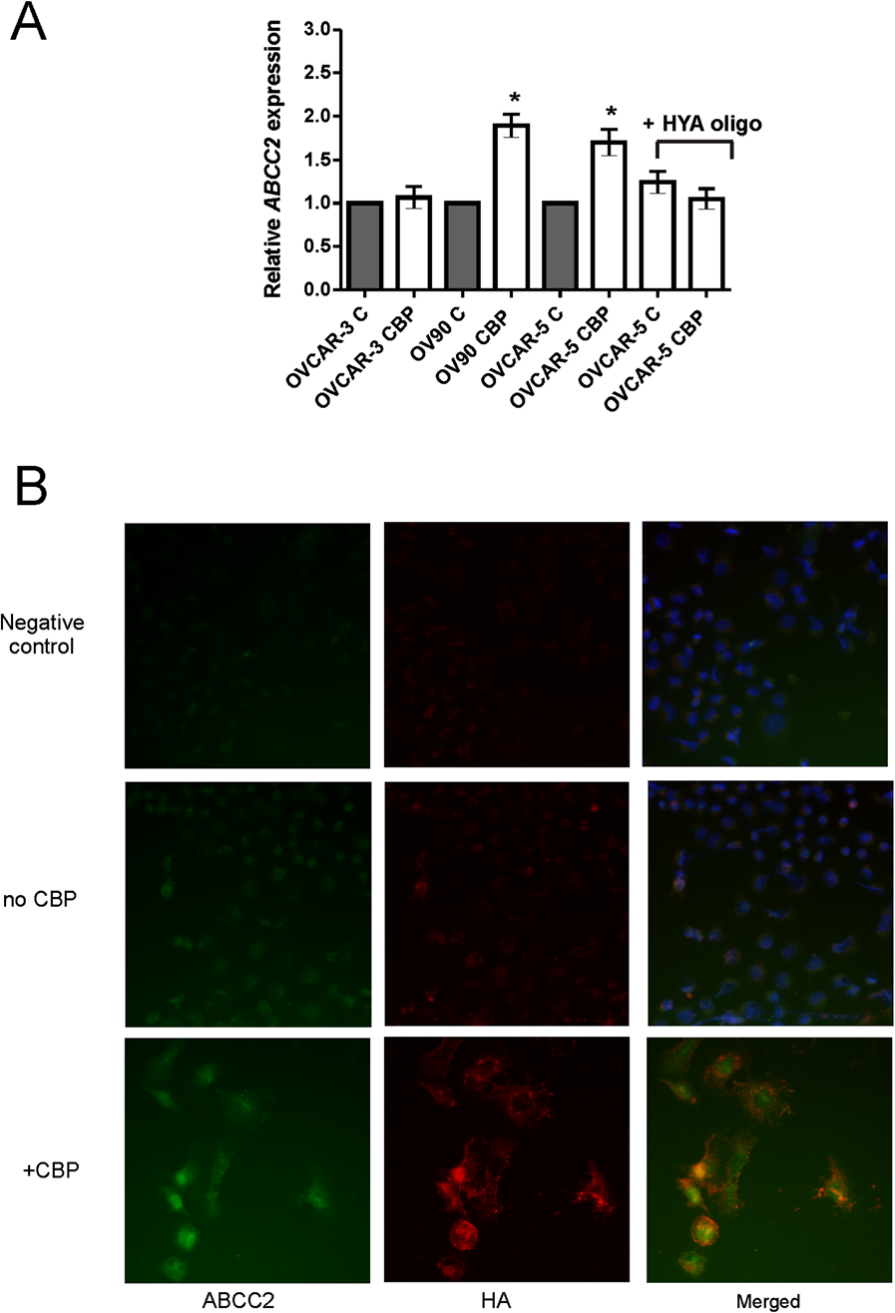
**Chemotherapy treatment increases ABCC2 expression in ovarian cancer cells. A**. qRT-PCR for *ABCC2* expression. Ovarian cancer cells treated with LD_50_ CBP for 72 hr. OVCAR-5 cells also treated in presence or absence of HA oligomers (HYA oligo, 250 μg/ml). Relative gene expression was determined by calibration against no CBP control and normalized to the house keeping gene β-actin using the 2^-∆∆CT^ method. Data represents mean ± SEM from 3 independent experiments performed in triplicate. **B**. OVCAR-5 treated with LD_50_ CBP for 72 hr. Fixed cells were incubated with biotinylated HABP and ABCC2 mouse monoclonal antibody and visualized with Cy3-conjugated streptavidin (red) and FITC-conjugated donkey anti-mouse immunoglobulins (green) respectively. Nuclei are counterstained with DAPI dye (blue).

Treatment with LD_50_ CBP dose significantly increased *ABCC2* expression in OV90 and OVCAR-5 cells but not OVCAR-3 cells. The increased *ABCC2* expression could be significantly blocked by the addition of HA oligomers in OVCAR-5 cells (Figure 4A). Increased HA and ABCC2 production in OVCAR-5 cells after exposure to CBP was confirmed by immunocytochemistry (Figure 4B). In the absence of CBP treatment, very little HA and ABCC2 were produced by OVCAR-5 cells (Figure 4B). CBP treatment increased cytoplasmic and extracellular HA and increased both cytoplasmic and nuclear ABCC2. HA and ABCC2 were found to be localized to the same cells (Figure 4B).

### HA is increased following chemotherapy treatment and predicts ovarian cancer outcome

**Figure 5:**
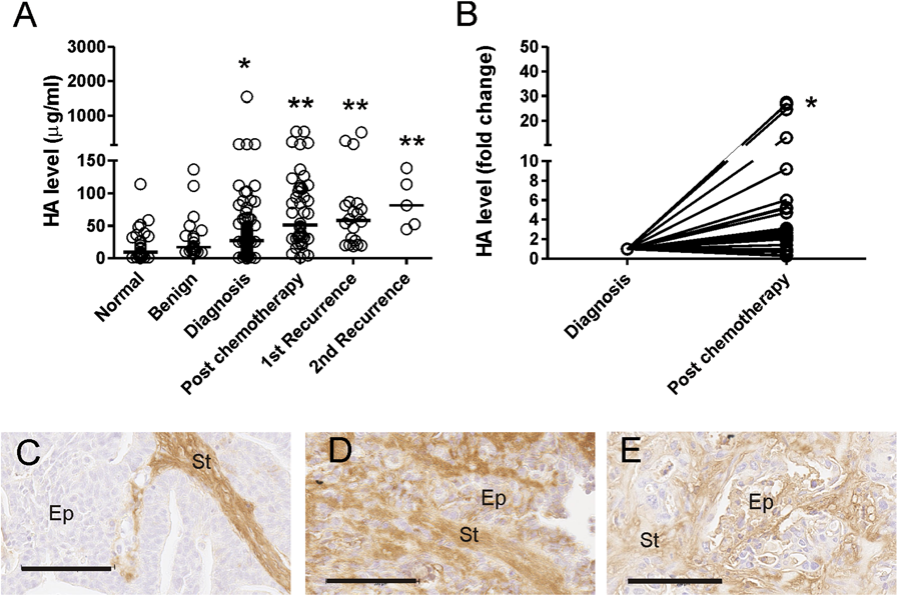
**HA is increased following chemotherapy treatment. A**. HA serum levels in ovarian cancer patients at diagnosis (cancer; n = 101), after at least 2 cycles of chemotherapy (post chemotherapy; n = 52), and at 1^st^ recurrence n = 17) and 2^nd^ recurrence (n = 5), as well as serum from patients with benign ovarian tumors (benign; n = 22) and from healthy controls (normal; n = 27). *, significantly different from normal control (*P* < 0.05, Mann Whitney U test). **, significantly different from HA serum level at diagnosis (*P* < 0.05, Mann Whitney U test). The bars specify the median values in each group. **B**. Fold change in serum HA levels in matched ovarian cancer patients at diagnosis and following chemotherapy treatment (n = 32). *, significantly increased from level at diagnosis (Wilcoxon rank test, *P* < 0.0001). HA staining in representative ovarian cancer tissues prior to treatment **(C)**, post chemotherapy **(D)** and at recurrence **(E)**. Ep = cancer cells, St = peritumoral stroma. Bar = 100 μm.

To determine whether chemotherapy can increase HA production in ovarian cancer patients, we measured serum HA levels at diagnosis, following chemotherapy, as well as at the time of recurrence. HA serum levels were also compared with those of patients with benign ovarian tumors and healthy controls. Serum HA levels were significantly higher in patients with ovarian cancer, when compared with normal controls (Figure 5A). HA levels were not significantly different between patients with ovarian cancer at diagnosis or benign ovarian tumors (Figure 5A), or between different ovarian cancer subtypes or stages of disease (Table 1). Serum HA levels were significantly increased in patients who received at least 2 cycles of chemotherapy (Figure 5A), and in patients at 1^st^ or 2^nd^ recurrence compared with serum HA levels at time of diagnosis (Figure 5A). Paired analysis found that serum HA levels were increased following chemotherapy in 75% (24/32) of patients (Figure 5B).

**Table 1:** HA levels in patients serum samples

**Tissue**		**n**	**Patient age (years)** **Median (range)**	**HA serum level (μg/ml)** **Median (range)**
**Normal ovaries**		27	50 (48–58)	9.6 (0.6–114.3)
**Benign ovarian tumors**		22	51 (35–62)	17.3 (1–136.5)
**Ovarian cancers**	**Total**	101	61 (25–90)	28.7 (0.3–1543.7)
	**Serous**	59	61 (25–85)	26.7 (0.3–172.8)
	**Endometrioid**	16	55 (33–90)	37.0 (4.2–1543.9)
	**Mucinous**	7	54 (37–74)	26.2 (1.5–70.7)
	**Clear Cell**	9	62 (37–82)	25.9 (2.2–101.9)
	**Others**	10	60 (32–88)	25.5 (13.6–111.9)
	**Stage**			
	1	31	55 (33–90)	26.0 (1.1–1543.7)
	2	6	59 (44–65)	19.0 (1.5–37.0)
	3	58	62 (25–88)	29.6 (0.3–172.8)
	4	4	64 (61–79)	19.8 (15.8–89.3)

We assessed HA levels in ovarian cancer tissues prior to treatment (at surgery) and in ovarian cancer tissues from patients that received neo-adjuvent chemotherapy and at recurrence to determine if HA production in ovarian cancer cells is increased following chemotherapy treatment. HA intensity in the cancer associated stroma was not different between the three tissue groups examined (*P* = 0.443, Pearson Chi Square). HA positive cancer cells were observed in 67% (6/9) of cancer tissues post chemotherapy (Figure 5D) and all recurrent cancer tissues examined (4/4, Figure 5E) but not observed in any of the serous ovarian cancer tissues obtained at surgery prior to any treatment (0/10, Figure 5C) (*P* = 0.001, Pearson Chi Square test).

**Figure 6:**
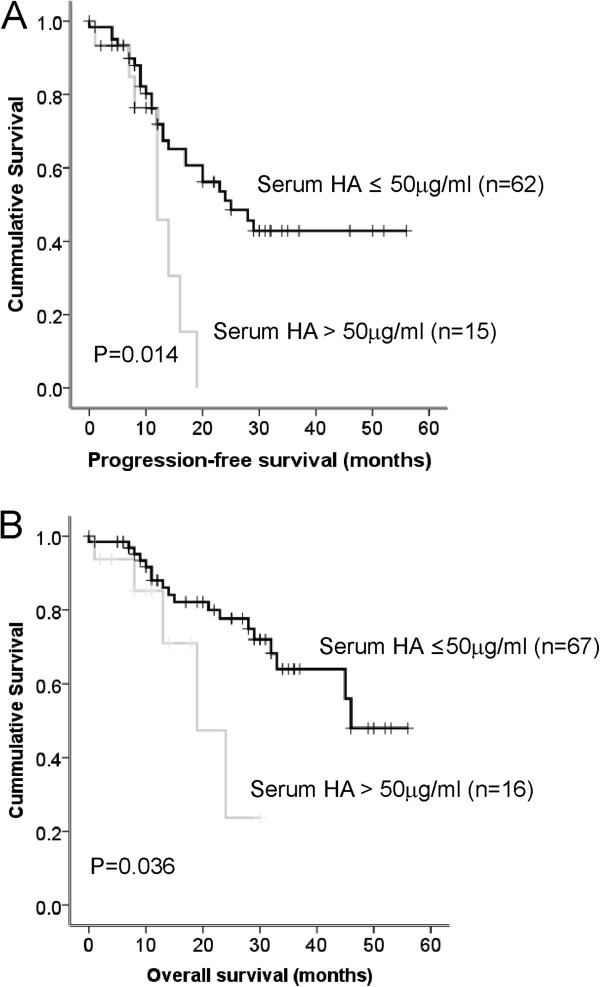
**Serum HA predicts ovarian cancer progression-free survival and overall survival. A**. Patients with high serum HA (> 50 μg/ml, n = 15) had a significantly reduced progression-free survival compared to patients with low serum HA (≤50 μg/mL, n = 62), log rank statistic = 6.035, *P* = 0.014. **B**. Patients with high serum HA (> 50 μg/ml, n = 16) had a significantly reduced overall survival compared to patients with low serum HA (≤50 μg/mL, n = 67), log rank statistic = 4.39, *P* = 0.036.

Kaplan-Meier analysis, using serum HA levels separated into quartiles demonstrated earlier relapses and deaths in the group of patients with serum HA levels > 50 μg/ml (fourth quartile) compared to patients in the other quartile groups (combined 1^st^-3^rd^ quartiles). The 12 month progression-free survival rate in patients with ≤50 μg/ml HA was 89.8% but only 45.8% in patients with serum HA levels > 50 μg/ml (*P* = 0.014, Figure 6A). Similarly the 2 year survival rate was 84% in the group of patients with HA ≤ 50 μg/ml and only 23.7% in the group of patients with HA > 50 μg/ml (*P* = 0.036, Figure 6B). Cox regression analysis showed that patients with serum HA levels > 50 μg/ml had a 2.7 fold (*P* = 0.02) increased risk of disease progression and 2.9-fold increased (*P* = 0.046) risk of ovarian cancer death. Due to cohort size, multivariate analysis was not performed in this study.

### HA regulates expression of ABC transporters

**Figure 7:**
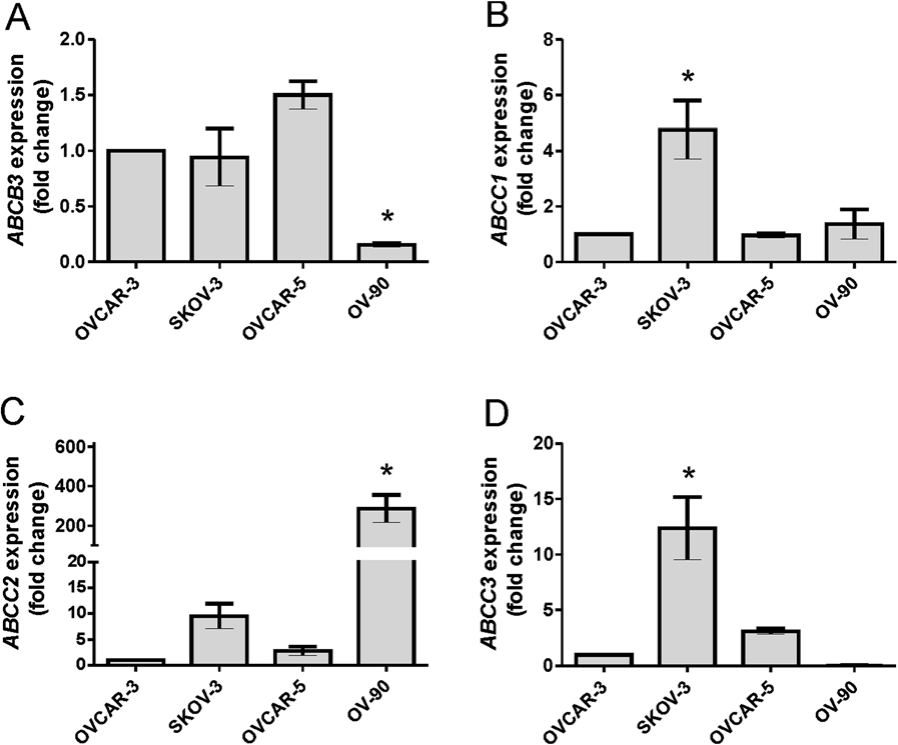
**ATP binding cassette (ABC) transporter expression by ovarian cancer cell lines.***ABCB3***(A)**, *ABCB4***(B)**, *ABCC2***(C)** and *ABCC3***(D)**. Relative gene expression was determined by calibration against OVCAR-3 cell expression and normalized to the house keeping gene β-actin using the 2^-∆∆CT^ method. Data represents mean ± SEM from 3 independent experiments performed in triplicate. *, significantly different from OVCAR-3 cells (*P* < 0.05, One Way ANOVA & Tukey multiple comparison post hoc test).

We compared relative expression levels of ABC transporter protein amongst the 4 ovarian cancer cell lines using the least invasive ovarian cancer cell line, OVCAR-3, as a calibrator (Figure 7A-D). We found highest expression of *ABCC1* (4.8 fold, Figure 7B) and *ABCC3* (12.4 fold, Figure 7D) in SKOV-3 cells, shown in Figure 1A to be the most resistant to CBP and to produce the highest level of HA (Figure 2A). Highest levels of *ABCC2* (286 fold) were observed in OV-90 cells (Figure 7C). *ABCB3* expression was lowest in OV-90 cells (Figure 7A) whilst *ABCB4* expression was only detected in OVCAR-5 cells (data not shown). We found no *ABCB1* expression in any of the ovarian cancer cell lines (data not shown). The MCF-7 breast cancer cell line, used as positive control for the real-time PCR experiments, expressed *ABCB1* (data not shown).

**Figure 8:**
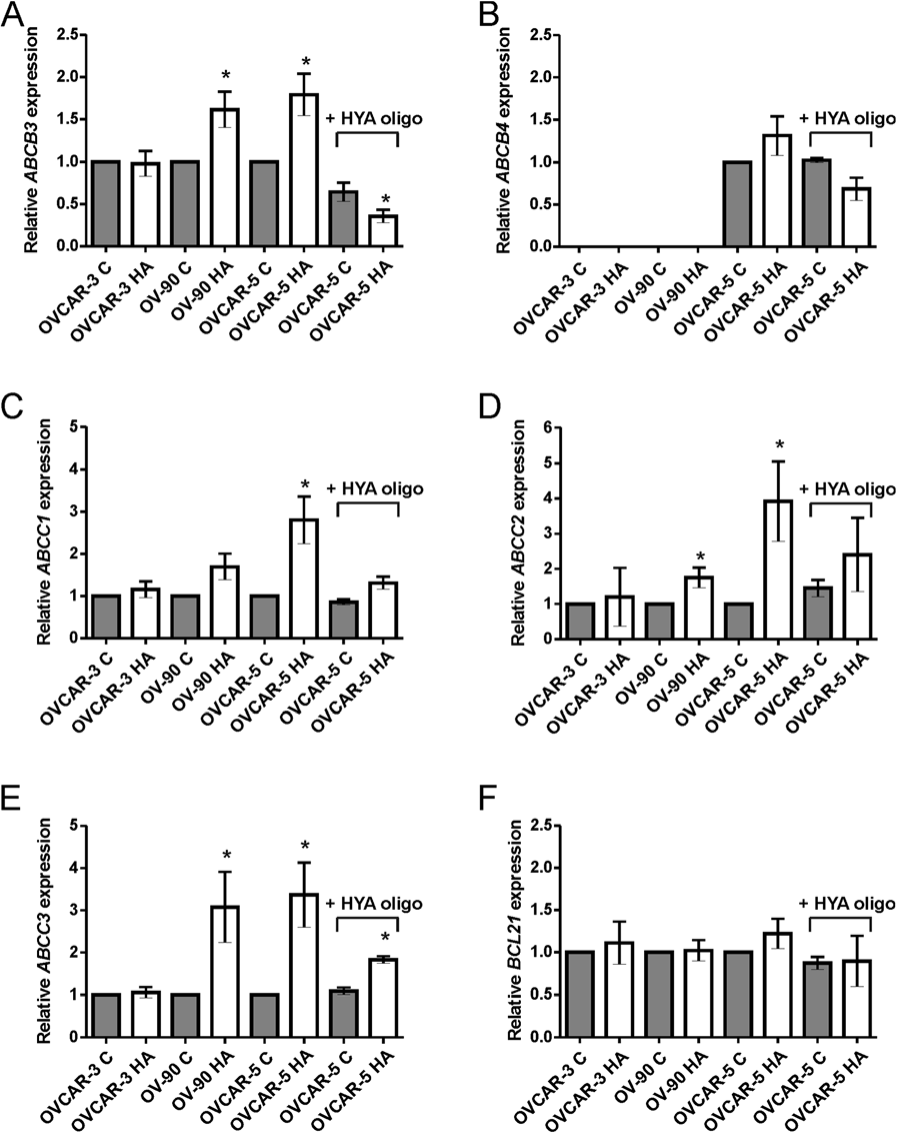
**HA regulates expression of ATP binding cassette (ABC) drug transporters. A**. *ABCB3*. **B**. *ABCC4*. **C**. *ABCC1*. **D**. *ABCC2*. **E**. *ABCC3* &**F**. *BCL21*. Ovarian cancer cells treated with HA (5 μg/ml) for 72 hr. OVCAR-5 cells also treated in presence or absence of HA oligomers (HYA oligo, 250 μg/ml). Relative gene expression was determined by calibration against no HA control and normalized to the house keeping gene β-actin using the 2^-∆∆CT^ method following 72 hr HA treatment. Data represents mean ± SEM from 2–4 independent experiments performed in triplicate following. *, significantly different from no HA control (*P* < 0.05, independent t test). C = no HA (control medium), HA = hyaluronan (5 μg/ml).

To further explore the molecular mechanisms of chemoresistance in ovarian cancer cells, we investigated whether HA treatment could regulate the expression of ABC transporters. HA (5 μg/ml) did not induce *ABCB1* expression in any of the ovarian cancer cell lines used in this study (data not shown). However, a significant increase in expression of *ABCB3* (1.87 fold, *P* = 0.004, Figure 8A), *ABCC1* (3.00 fold, *P* = 0.012, Figure 8C), *ABCC2* (3.18 fold, *P* = 0.002, Figure 8D), and *ABCC3* (3.40 fold, *P* = 0.003, Figure 8E) was observed in OVCAR-5 cells treated with HA (5 μg/ml) for 72 hours. *ABCB3* (1.61 fold, *P* = 0.028, Figure 8A), *ABCC2* (1.75 fold, *P* = 0.039, Figure 8C) and *ABCC3* (3.08 fold, *P* = 0.048, Figure 8E) expression was also significantly increased in OV-90 cells following HA treatment. *ABCB4* expression in OVCAR-5 cells was not altered by HA treatment (Figure 8B). No significant change in ABC transporter expression was observed in HA treated OVCAR-3 cells (Figure 8A-E). The addition of HA oligomers (250 μg/ml) completely abrogated the increase in expression of *ABCB3* (Figure 8A), *ABCC1* (Figure 8C), *ABCC2* (Figure 8D), and partially blocked *ABCC3* expression in OVCAR-5 cells (Figure 8E). The expression of the anti-apoptotic gene *Bcl-x*_*L*_ was not altered by HA treatment in any of the cell lines (Figure 8F).

## Discussion

Chemotherapy resistance is one of the most challenging problems in cancer treatment. The molecular mechanisms mediating chemoresistance are widely studied but still poorly understood. Multidrug transporter proteins, such as ABC transporters, are well known for their contribution to chemoresistance through the efflux of cytotoxic drugs from cancer cells [40,41]. ABC transporter expression and chemoresistance has been reported to be modulated by HA-CD44 interactions [30,32-34,42]. Although the importance of HA and CD44 in ovarian cancer progression has been well established [18,25,43], the knowledge about their significance in mediating ovarian cancer chemoresistance is limited.

Our results show that HA induces chemoresistance against CBP, and increases the expression of the ABC transporters, *ABCB3*, *ABCC1*, *ABCC2*, and *ABCC3* in ovarian cancer cell lines expressing the HA receptor, CD44. By measuring serum HA levels in ovarian cancer patients, we demonstrate for the first time that HA levels are elevated in patients following chemotherapy treatment and at recurrence compared with HA levels at diagnosis. Importantly, higher serum HA levels (> 50 μg/ml) were associated with reduced progression-free and overall survival. Our findings confirm that, in addition to their important role in promoting malignant ovarian cancer cell behaviour, CD44-HA interactions also play a significant role in mediating chemoresistance.

HA production in ovarian cancer cells was increased in ovarian cancer tissues from patients that received neoadjuvent chemotherapy and at recurrence compared to tissues collected at surgery prior to any treatment. Other potential mechanisms that may also contribute to the increased HA serum levels following chemotherapy were not investigated further in this study. Pro-inflammatory cytokines including tumour necrosis factor and interleukin-1β shown to be increased by chemotherapy treatment [44,45] and stimulate HA production in a variety of cell types [46-49] may also contribute to increasing serum HA levels.

Our data supports the model that chemoresistance is acquired by chemotherapy-induced HA production and increases ovarian cancer cell survival which contributes to chemoresistance by increasing the expression of ABC transporter proteins via a HA-CD44 mediated pathway. CBP treatment increased the secretion of HA in ovarian cancer cell lines by increasing HA synthesis, as we found corresponding increased *Has2* and *Has3* expression in OVCAR-5 and increased *Has3* expression in OV-90 and OVCAR-3 cells. In accord with our results, increased chemotherapy resistance was observed in MCF7 breast cancer cells overexpressing *Has2*[50]. Furthermore, chemoresistant lymphoma cell lines were also found to have greater expression of *Has1*, *Has2*, and *Has3* transcripts, and to secrete higher levels of HA [51].

Our finding that HA promotes chemoresistance of ovarian cancer cells agrees with previous studies using other cancer cell lines showing that HA can increase the LD_50_ of various chemotherapy drugs [26-29,31]. We have found that the addition of either neutralizing CD44 antibody or HA oligomers (6–10 sugar residues), which interact monovalently with CD44 and competitively block polyvalent interactions between CD44 and endogenous HA, blocked the HA induced chemoresistance in CD44 positive ovarian cancer cell lines but not in the CD44 negative, OVCAR-3 cells. These findings demonstrate the significance of HA-CD44 interactions in this mechanism. Furthermore, HA oligomers were able to sensitize chemoresistant SKOV-3 cells to CBP. These findings agree with previous studies demonstrating that HA oligomers can sensitize various carcinoma cell lines to chemotherapy drugs, including doxorubicin, taxol, and vincristine, both *in vitro* and *in vivo*[30,32,34]. More recently, Slomiany *et al.* found that HA oligomers decreased doxorubicin resistance of malignant peripheral nerve sheath tumours and suppressed HA secretion in these tumors [52]. Importantly, the HA oligomers and doxorubicin acted synergistically at suboptimal doses and induced tumour regression to a greater extent than either agent alone [52].

Several studies have also shown that HA-CD44 interactions regulate ABC transporter expression and activity [28,30-32]. HA has been shown to stimulate *ABCB1* expression and *ABCB1* activity in various cancer cell lines [28,30-32]. HA-CD44 interactions also regulate expression of *ABCG2* (BCRP) in glioma cells [34]. *ABCC2* expression is upregulated in non small cell lung cancer cells (H322) overexpressing CD44, which are more resistant to cisplatin when cultured on a HA matrix [33]. Human mesenchymal stem cells cultured on a layer of HA are more resistant to doxorubicin and produce increased levels of *ABCB1*[53]. Interestingly, we found that SKOV-3 cells, which produced the highest HA levels, were most resistant to CBP and expressed the highest levels of *ABCC1* and *ABCC3*. CD44 co-localizes in the plasma membrane of cancer cells with *ABCB1* and *ABCG2*, and HA antagonists rapidly induce internalization of these transporters and CD44 to make them ineffective [34,52,54]. These findings have led to the suggestion that multivalent interactions between HA and CD44 may be necessary for stabilization of transporter interactions within the plasma membrane [52].

Bourguignon *et al.* have previously reported *ABCB1* expression in SKOV-3.ipl, a variant cell line established from mouse xenograft, to be increased by HA treatment [28]. However, we did not observe *ABCB1* expression in any of the ovarian cancer cell lines examined, neither in the absence nor presence of HA. In contrast to Bourguignon *et al.* 2009 [31], we did not observe an effect on *Bcl-2l* expression by HA treatment in the ovarian cancer cell lines. In our experiments, HA alone had no effect on cell survival and an anti-apoptotic mechanism was excluded. Our findings suggest that HA induces chemoresistance by inducing ABC transporter expression, which increases ovarian cancer cell survival by increasing the efflux of CBP from the cells.

Our data supports a role for *ABCB3*, *ABCC1*, *ABCC2*, and *ABCC3* in ovarian cancer chemoresistance, which is in agreement with previous studies. Overexpression of *ABCC1* and *ABCC2* in human ovarian cancer cells conferred marked resistance to chemotherapeutics, such as methotrexate [55]. Furthermore, increased expression of *ABCC1* was observed in ovarian cancers from chemotherapy non-responders [56]. Higher gene expression of *ABCC1* and *ABCC3* was also found in ovarian cancer patients with unfavourable outcome following debulking surgery and platinum based chemotherapy [12]. Nuclear membrane localization of *ABCC2* has been shown to predict resistance to cisplatin [39]. A recent study by Auner *et al.* identified gene expression of the four ABC transporters *ABCC1*, *ABCC2*, *ABCC3* and *ABCB3* to be significantly elevated in recurrent ovarian cancer compared to benign tumors and untreated primary cancer [15]. Oxaliplatin resistant ovarian cancer cells have been shown to overexpress *ABCC1* and *ABCC4*[57]. The importance of *ABCC2* in mediating chemoresistance is supported by our findings showing increased expression of *ABCC2* in ovarian cancer cells following treatment with CBP and verified by studies which showed that *ABCC2* siRNA knockdown could reverse cisplatin and paclitaxel resistance in ovarian cancer cell lines [39,58].

HA is a promising candidate for increasing efficacy and reducing toxicity of cancer therapies. Administration of HA-chemotherapy conjugates, which allows chemotherapy drugs to enter cancer cells via a CD44 receptor-mediated mechanism, resulted in increased therapeutic activity compared with chemotherapy alone [59,60]. In the taxane resistant HeyA8-MDR ovarian cancer xenograft model, metronomic doses (more frequent lower therapeutic doses) of paclitaxel-HA conjugate had a more effective anti-tumor activity and exhibited reduced toxicity compared with mice that were administered the maximum tolerated doses of the paclitaxel-HA conjugate [61]. More recent studies have demonstrated that HA is a barrier for chemotherapy drugs in pancreatic cancers [62,63]. Systemic administration of chemotherapy together with hyaluronidase (PEGPH20), which degrades HA, improved blood vessel perfusion and resulted in increased sensitivity and improved survival in pancreatic cancer mouse models [62,63]. Clinical trials are ongoing to examine the effect of depleting HA in patients with pancreatic cancer (NCT01959139, NCT01839487 & NCT01453153, http://clinicaltrials.gov). Together these studies and our recent findings support the notion that targeting HA in the tumor microenvironment is an important future direction for cancer therapy.

## Conclusions

This study has demonstrated that HA treatment reduces the ability of CBP to cause cell death and increases the expression of ABC transporters in CD44 positive ovarian cancer cell lines. Importantly, we also found that serum HA levels are increased in ovarian cancer patients following chemotherapy treatment and at recurrence. These findings support the theory that chemoresistance is acquired as a response to chemotherapy and the increased production of HA can contribute to chemoresistance by regulating expression of ABC transporters via a HA-CD44 mediated pathway. Targeting the HA-CD44 signaling pathway is therefore a promising strategy to overcome chemoresistance and improve ovarian cancer survival.

## Abbreviations

ABC: ATP binding cassette; CBP: Carboplatin; CM: Conditioned media; DAPI: 4′,6′-diamidino-2-phenylindole dihydrochloride solution; ECM: Extracellular matrix; FBS: Fetal bovine serum; HA: Hyaluronan

## Competing interests

The authors declares that they have no competing interests.

## Authors’ contributions

CR, MPW and MKO conceived the idea and designed the experiments; CR, MPW, NAL, IAT and CEP performed the experiments. All authors read and approved the manuscript.

## Pre-publication history

The pre-publication history for this paper can be accessed here:

http://www.biomedcentral.com/1471-2407/13/476/prepub

## Supplementary Material

Additional file 1: Table S1 TaqMan primers used for gene expression studies. Click here for file

Additional file 2: Figure S1 Effect of HA on ovarian cancer cell proliferation. Ovarian cancer cells treated with increasing concentration of HA (0–500 μg/ml) for 72 hr. The highest concentration of HA (500 μg/ml) inhibited the growth of OVCAR-5 and SKOV3 cells. Data are expressed as percentage control, mean ± SEM from 3 independent experiments performed in triplicate. *, significantly different from control (*P* < 0.05, independent t test). Click here for file
