# Supplementary material for: Chemotherapy-induced hyaluronan production: a novel chemoresistance mechanism in ovarian cancer
Source: BMC Cancer. 2013 Oct 14;13:476. doi: 10.1186/1471-2407-13-476 (PMC3852938; doi:10.1186/1471-2407-13-476)
Supplement: Additional file 1: Table S1 — TaqMan primers used for gene expression studies. [file 1471-2407-13-476-S1.docx]

**Additional file 1: Table S1. TaqMan primers used for gene expression studies**

| **Gene** | **Assay ID** |
| --- | --- |
| *Has2* | Hs00193435_m1 |
| *Has3* | Hs00193436_m1 |
| *ABCB1* | Hs01067802_m1 |
| *ABCC1* | Hs00219905_m1 |
| *ABCC2* | Hs00166123_m1 |
| *ABCC3* | Hs00978473_m1 |
| *ABCB3* | Hs00241060_m1 |
| *ABCB4* | Hs00240956_m1 |
| *BCL2L1* | Hs00236329_m1 |
