# Supplementary figures and images for: Chemotherapy-induced hyaluronan production: a novel chemoresistance mechanism in ovarian cancer
Source: BMC Cancer. 2013 Oct 14;13:476. doi: 10.1186/1471-2407-13-476 (PMC3852938; doi:10.1186/1471-2407-13-476)

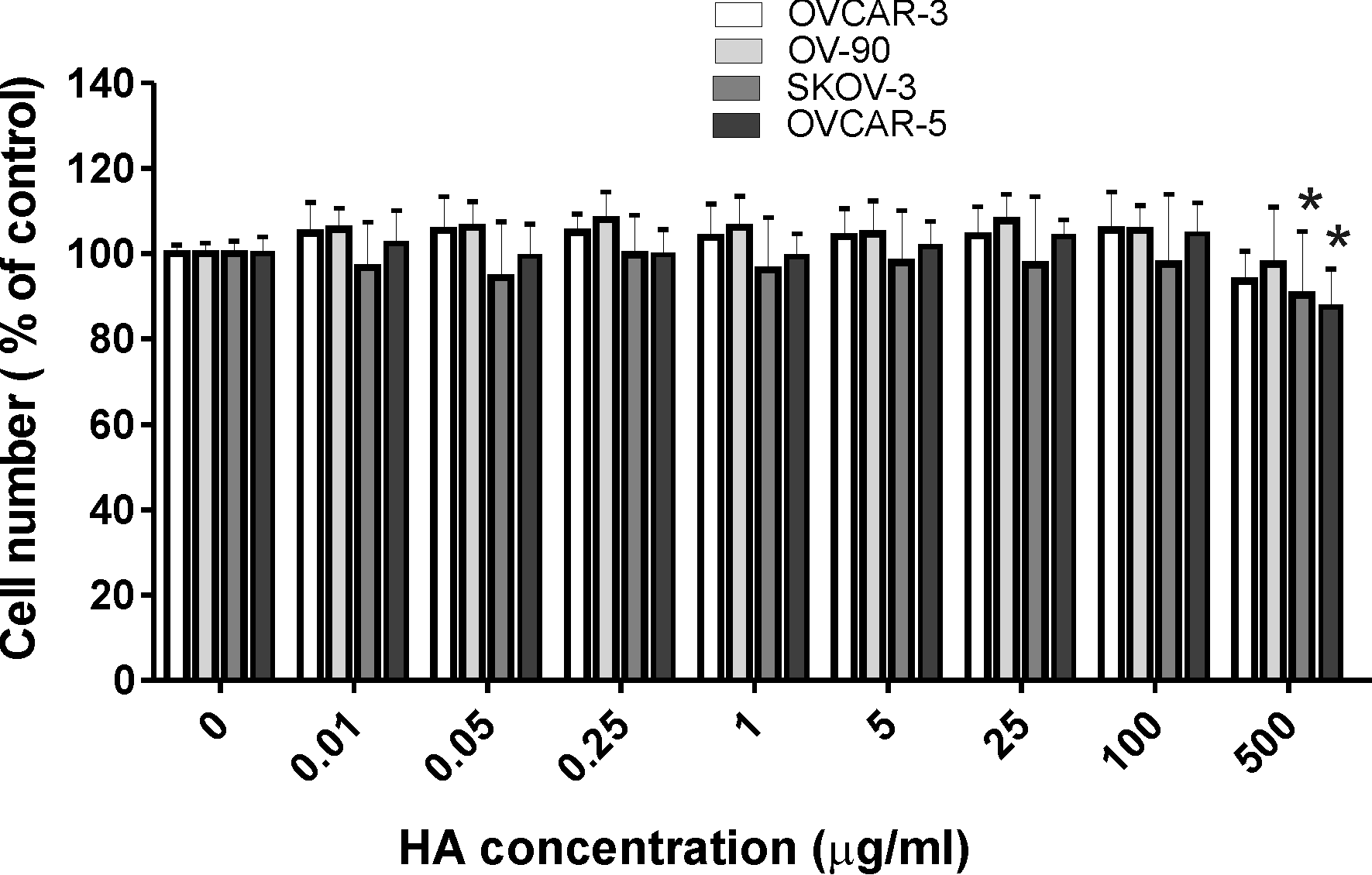

Supplement: Additional file 2: Figure S1 — Effect of HA on ovarian cancer cell proliferation. Ovarian cancer cells treated with increasing concentration of HA (0–500 μg/ml) for 72 hr. The highest concentration of HA (500 μg/ml) inhibited the growth of OVCAR-5 and SKOV3 cells. Data are expressed as percentage control, mean ± SEM from 3 independent experiments performed in triplicate. *, significantly different from control (P < 0.05, independent t test). [file 1471-2407-13-476-S2.tiff]
